# Development of novel biocompatible thermosensitive anti-adhesive agents using human-derived acellular dermal matrix

**DOI:** 10.1371/journal.pone.0212583

**Published:** 2019-02-22

**Authors:** Jong Ju Jeong, Dong Won Lee, Seung Yong Song, Yerin Park, Ji Hee Kim, Jang Il Kim, Hyung Goo Kim, Ki Taek Nam, Won Jai Lee, Kee-Hyun Nam, Ju Hee Lee

**Affiliations:** 1 Department of Surgery, Yonsei University College of Medicine, Seoul, Republic of Korea; 2 Department of Plastic and Reconstructive Surgery, Institute for Human Tissue Restoration, Yonsei University College of Medicine, Seoul, Republic of Korea; 3 R&D Center, L&C BIO Co., Ltd, Gyeonggi-do, Republic of Korea; 4 Department of Dermatology and Cutaneous Biology Research Institute, Yonsei University College of Medicine, Seoul, Republic of Korea; 5 Severance Biomedical Science Institute, Yonsei University College of Medicine, Seoul, Republic of Korea; Shanghai Jiao Tong University Medical School Affiliated Ruijin Hospital, CHINA

## Abstract

Postoperative adhesion is a natural phenomenon that occurs in damaged tissue cells. Several anti-adhesion agents are currently used, but there is no leading-edge product with excellent adhesion-preventive effects. The purpose of this study was to develop ideal anti-adhesive agents using human-derived acellular dermal matrix (ADM). We developed 5 new biocompatible thermosensitive anti-adhesion barriers (AABs) using micronized human-derived ADM, hyaluronic acid, and temperature-sensitive and biocompatible synthesized polymers. The biocompatibility, anti-adhesion effect, and biodegradability of these AABs were compared with those of commercial thermosensitive anti-adhesion agents. No cytotoxic effects were observed in vitro and in vivo. Animal testing of adhesion resistance confirmed that the adhesion area, strength, and grade of AAB03 were statistically superior to those of the control group. Factors related to adhesion formation, such as lymphocytes, macrophages, microvessels, and collagen fiber density, were observed using specific staining methods; the results confirmed that AAB03 group exhibited significantly lower macrophage counts, microvessel density, and collagen fiber density than the control groups. Furthermore, AAB03 was completely absorbed by 6 weeks. Thus, AAB03 has the potential to be used as a high-performance anti-adhesion agent.

## Introduction

Postoperative adhesions refer to the fibrous bands that form between tissues and organs because of a surgical procedure, and they are a natural phenomenon occurring during the proliferation and regeneration of damaged tissue cells. However, excessive adhesions or adhesions in other unintended organs or tissues can lead to organ malfunction, which may require surgical reintervention for detachment of the adhesions and is potentially life-threatening [1]. Moreover, postoperative tissue adhesions can cause intestinal obstruction, chronic pain, sexual dysfunction, and sterility [2,3]. According to Ouaïssia et al. [4], peritoneal adhesions after abdominal surgery cause 32% of all acute intestinal obstructions and 65%-75% of all small bowel obstructions. Furthermore, peritoneal adhesions develop in 93%-100% and 67%-93% of upper and lower abdominal laparotomies, respectively; of these, 15%-18% require surgical reintervention. Although laparoscopic surgery is associated with a reduced adhesion incidence rate, adhesions occur in approximately 45% of all laparoscopic surgeries [5].

As the average lifespan has increased, concerns regarding not only treatment effectiveness, but also postoperative sequelae have become serious. Postoperative sequelae can degrade the quality of life. For example, patients with a good prognosis who develop dysphagia after thyroid surgery live with swallowing difficulty [6]. Furthermore, contractures due to postoperative adhesions may force patients to live with external shrinkage, causing psychological distress and inconvenience for the rest of their lives. Additionally, surgical procedures to remove postoperative adhesions are becoming an economic and physical burden. Mais et al. [7] reported that 967,332 days and 2.25 billion dollars were spent treating adhesions in patients in the United States of America (US) in 2005 [7]. Although the number of cases of postoperative adhesions and the cost of their treatment in South Korea are undetermined, they are assumed to be comparable to those in the US.

Current methods of adhesion prevention can be broadly classified as: (1) minimizing damage to tissues from unnecessary procedures and adhesions due to foreign material reactions [8]; (2) suppressing inflammatory reactions and pathophysiological processes necessary for the formation of adhesions through the use of drugs targeting adhesion mechanisms; and (3) blocking contact with surrounding tissues by wrapping or covering wound regions with anti-adhesion barriers (AABs). Furthermore, laparoscopy or minimally invasive surgical procedures are helpful for the prevention of adhesions by minimizing or preventing trauma, exposure to foreign materials, and tissue drying and promoting hemostasis via their effects on the pneumoperitoneum [9]. Although these methods can reduce the formation of adhesions, they cannot completely prevent adhesions. As the prevention of postoperative adhesions is crucial, careful surgical procedures and the use of high-performance anti-adhesive agents should be considered.

Anti-adhesive agents include fibrinolytic agents [10], which prevent the formation of adhesions, anticoagulants [11], anti-inflammatory drugs [12], antibiotics for the prevention of infections, and other hormone drugs [13]. Currently used AABs include film/membrane, solution, and gel types [14–16]. Film/membrane anti-adhesive agents are associated with reduced adhesiveness when applied to internal organs and foreign body reactions, the folding and sticking properties of the film can cause application difficulties during minimally invasive surgical procedures or laparoscopy, and adhesion may occur in sutured regions [17]. Solution-type anti-adhesive agents have limited adhesion prevention, as in many cases they cannot be accurately applied to wounds because the solution flows down the body or to other regions and can decompose rapidly [18]. Finally, gel–type anti-adhesive agents have limited adhesion preventive effects because they do not remain in the wound tissue for a sufficient amount of time, as they dissolve and are discharged before wound healing, and are associated with foreign body reactions in vivo when comprised of non–in-vivo–derived substances [19].

Currently, there is no leading-edge product available with excellent adhesion-preventive effects. Therefore, the purpose of the present study was to develop a better anti-adhesive agent that can help prevent adhesions, thereby reducing additional expenses due to postoperative adhesion management and improving the quality of life of patients. Using both in vitro and in vivo methods, we initially tested 5 candidate AABs, comparing their properties to those of anti-adhesive agents commercially available in South Korea, with further testing performed in the most promising AAB candidate(s).

## Materials and methods

### In vitro study

#### Platform preparation of new AAB candidates

Micronized ADM was manufactured using ADM (MegaDerm^®^; L&C BIO, Seongnam-Si, Gyeonggi-Do, Korea) derived from donated human skin supplied by US tissue banks under the guidelines of the American Association of Tissue Banks (AATB) and the US Food and Drug Administration. First, the ADM was cut into squares of approximately 1 cm, using a surgical blade. Then, 100 g of ADM was placed in a microgrinder (IKA MF10; IKA Works Inc., Wilmington, USA) that would not destroy the tertiary structure of the ADM and was ground at 5000 rpm for 3 minutes, as this provides the optimal conditions for preventing collagen and elastin denaturalization. The ground ADM was filtered using a 500-μm sieve under aseptic conditions, yielding micronized ADM that exhibits optimal retention. Five types of AAB samples (AAB01, AAB02, AAB03, AAB04, and AAB05) were manufactured following the procedure described in Table 1.

**Table 1:** Formulations of the prepared and commercial anti-adhesion agents.

	PF-127wt%	HA % (w/v)	Micronized ADM % (w/v)	Viscosity at 25°C Pa·s	Viscosity at 37°C Pa·s
**CTL1**	25~35	0	0	1.282	35.681
**CTL2**	N/A	0	0	2.0≤	15.0≤
**AAB01**	16.0	0.2	3.5	4.535	17.560
**AAB02**	17.0	0.2	1.75	6.057	30.570
**AAB03**	18.0	0.2	1.75	10.554	38.487
**AAB04**	19.0	0.1	1.75	27.960	30.304
**AAB05**	20.0	0.1	1.75	36.991	26.658

PF-127: poloxamer 127 (Kolliphor^®^ P407; BASF, Ludwigshafen, Germany), HA: hyaluronic acid (Shiseido sodium hyaluronate SZE grade-EP; Shiseido, Tokyo, Japan), ADM: human-derived acellular dermal matrix (ADM; MegaDerm^®;^ L&C BIO Co., Ltd/R&D Center, Gyeonggi-do, South Korea) [20,21], AAB: anti-adhesion barrier, CTL1: commercial anti-adhesion agent 1, CTL2: commercial anti-adhesion agent 2

Briefly, three 10-mL Luer lock syringes (syringe 1, 2, and 3) were prepared. Poloxamer and hyaluronic acid were placed in syringe 1 and vortexed. Syringe 2, which contained 10 mL of cold distilled water, was connected to syringe 1 using a connector and the contents were mixed. After the poloxamer/hyaluronic acid powder was completely hydrated, the syringe was connected to syringe 3, which contained micronized ADM, and the contents were mixed. The syringe was stored upright at 4°C. After removing the bubbles, the layered AAB was additionally mixed, and the bubbles were again removed at 4°C. Finally, the prepared AAB was sterilized using gamma rays (25 kGy).

#### Assessment of temperature-dependent viscosity

Temperature-dependent changes in the viscosity of the five AAB candidates and a commercial anti-adhesion agent 1 (CTL1) were measured using a rotational rheometer (DHR-1; TA Instrument Ltd., New Castle, DE, USA). Viscosity was measured within a temperature range of 25°C-50°C, using a disposable 25-mm plate, at a constant shear rate of 10 1/s and heating rate of 1°C/min to determine the thermosensitivity of the AAB candidates, which was compared to that of CTL1.

#### In vitro gel stability test

Based on the thermosensitivity and composition assessment results, two milliliters of three AAB candidates (AAB01, AAB02, AAB03) were placed in separate 5-mL glass test tubes. The test tubes were sealed and left standing at 37°C for 30 minutes to convert the contents to gel. After adding 2 mL of RPMI media, the test tubes were centrifuged at 50 rpm and 37°C for 4 days. The AAB that dissolved through RPMI infiltration was removed every 24 hours, and 2 mL of fresh RPMI was added. The height of the residual gel was measured at 24, 48, 72, and 96 hours to compare the stability of the gel with that of CTL1.

#### Cytotoxicity test (ISO 10993–5: Extraction method and agar diffusion test)

Elution of the most ideal candidate substance, AAB03, was tested by completely solidifying 4 g of the test substance into gel at 37°C. Twenty mL of RPMI1640 medium containing 10% Fetal bovine serum (FBS) and 1% Penicillin-streptomycin (P/S) and an amount of media equivalent to the absorbent capacity of the substance (1.3 mL) were added and incubated in a CO incubator for 24 hours at 37°C. The eluate was centrifuged at 3000 rpm at room temperature (25°C) for 10 minutes and filtered with a 0.45-μm syringe. Mouse fibroblast cells (L 929; ATCC®-CCL-1^™^) were obtained from the Korean Cell Line Bank. The cells were subcultured once using RPMI1640 (10% FBS, 1% P/S) media before the experiment. The subcultured cells were seeded in a six-well plate at 2×10^5^ cells/2 mL/well. After culturing for 24 hours, 2 mL of the eluates for each sample were added to each well, and cell morphology and viability were examined 48 hours later. This experiment was performed three times. Cell morphology was examined using an optical microscope (Olympus, CKX41-A32PHP, Japan) at x100 and x400 magnification. Cell count was graphed by computing cell viability against that of the reagent control. Based on the results, the cytotoxicity grade was predicted by referencing the ISO standard.

For the agar diffusion test, 1% agar media was treated using the sample and the grade was predicted as per the ISO standard after 24 h. Briefly, the standard agar diffusion assay was adapted to tissue culture dishes; 1×10^5^cells/ml was poured into an empty dish and grown to confluence for 24 h at 37°C. The supernatant culture medium was then poured off and replaced with fresh agar medium. After an agar layer formed, neutral red solution was added to the dish. The vital stain solution was then poured off and the flat surface of the test specimen was placed on the agar surface. Two sample disks were tested, along with positive and negative controls, in the same plate. After incubation, the samples were carefully removed to detect zones of decolorization (in mm) and cell lysis.

### In vivo study

#### Animal protocol and surgical technique

The in vivo experimental protocol was approved by the Institutional Animal Care and Use Committee at Yonsei University (IACUC approval no. 2016–0018). Male Sprague-Dawley rats (250–300 g; Samtaco Bio, Osan, South Korea) were raised in the species pathogen-free area of ABMRC at Yonsei University Health System (AAALAC Full Accreditation, #001071). After 1 week of acclimatization, ten 6-week-old rats were divided into four groups (no treatment, CTL1, CTL2, and AAB03) for each experiment. The rats were anesthetized with Zoletil 30 mg/kg, Rompun 10 mg/kg IM, or Avertin 300 mg/kg IP to obtain abdominal adhesion models. The abdomen was shaved, swabbed with Povidine, and cut 4–5 cm along the central line. The peritoneal epithelium (1 × 1 cm) was cut 2 cm away from the incision on the abdomen. To induce abrasion, the surface of the appendix touching the wound in the peritoneum was rubbed with sterilized sandpaper (800 Cw, 1 × 4 cm; Diamond, Seoul, South Korea) until bleeding and ruptured capillaries were observed. Then, no treatment or 1 mL of the experimental substances (CTL1, CTL2, and AAB03) was applied to the abrasion, and the abdominal wall was sutured. The test animals were sacrificed 7 days later by CO_2_ gas inhalation.

To assess absorptivity, CTL1, CTL2, and AAB03 were subcutaneously injected dorsally into the adhesion model (n = 10). After sacrificing the animals 7days post-injection, the number of animals with residual sample was determined. The absorption rate was calculated as 1-(the number of animals with residual sample/ total number of animals) x100 (%). To confirm the final absorption time, the samples were subcutaneously injected in four areas among the three groups of twelve 6-week-old rats in each experiment. After the injection, two rats from each group were sacrificed every week to measure the residual sample until week 6 (S1 Fig).

Different organs (brain, heart, kidney, liver, and spleen) were harvested and fixed in 4% paraformaldehyde in phosphate-buffered saline (PBS) for systemic toxicity assessment (n = 10). These tissues were sectioned, stained with hematoxylin and eosin (H&E), and observed in a blinded manner (S2 Fig).

#### Assessment of anti-adhesive effects

The sacrificed animals in the four groups (no treatment, CTL1, CTL2, and AAB03) were dissected to confirm the area, strength, and grade of adhesion using a blinded method (S3 Fig). The adhesion grades were classified into 3 grades based on the Hooker’s score (0, no adhesion; 1, gentle blunt dissection required to free adhesion; 2, aggressive blunt dissection required to free adhesion; and 3, sharp dissection required to free adhesion) or 4 grades based on the Blauer’s score (0, no adhesions; 1, thin or narrow, easily separable adhesions; 2, thick adhesions limited to one area; 3, thick and widespread adhesions; and 4, thick and widespread adhesions, plus adhesions of the viscera to anterior and/or posterior abdominal wall), as described elsewhere [22,23]. The adhesion area was calculated by measuring the major and minor axes of the adhesion region. The adhesion strength was defined using the following scale: 1, adhesion was filmy and easily torn with very light pressure; 2, adhesion was substantial and needed moderate pressure to tear; 3, adhesion was heavy and required significant pressure to rupture; and 4, adhesion was very heavy and was difficult to rupture [24].

#### Histological and immunohistochemical analysis

After making incisions on the abdomens of the sacrificed animals (n = 10), we isolated the areas exhibiting severe adhesions and fixed them in 10% formalin for 24 hours to prepare paraffin blocks. Subsequently, 4–5-μm sections were prepared from the blocks to facilitate identification of the factors related to adhesion. The presence of cells was assessed using H&E staining. The degree of collagen production was assessed by measuring the collagen fiber density using Masson’s trichrome staining. Collagen fiber density was determined using Image J software (NIH, Bethesda, MD). Values for the total percentage per x400 field of smooth muscle‐collagen were then calculated. Immunohistochemical analysis was implemented to assess the infiltration of inflammatory cells and microvessel formation. Lymphocytes were examined using immunohistochemical staining with CD3 antibody, a T-lymphocyte marker (dilution = 1:100) and measured by counting the number of CD3+ cells per total cells stained with hematoxylin. Macrophages were counted using an optical microscope after immunostaining with antibodies for ED1 and F4/80, which are pan and murine-specific macrophage markers (dilution = 1:100 and 1:50, respectively). The microvessel density was measured using immunostaining with CD31 antibody, an endothelial cell marker (dilution = 1:100). The extent of vessel formation within the adhesion was analyzed by counting the number of vessels using a fluorescent microscope (Olympus BX53, Olympus Corporation, Tokyo, Japan) and measuring the microvessel density.

### Statistical analysis

Data are expressed as means ± standard error (SE). Differences among treatments were evaluated using the Kruskal–Wallis rank test and post-hoc comparisons (Scheffe and Steel-Dwass). Values of p<0.05 were considered statistically significant. All statistical analyses were performed using IBM SPSS software version 23.0 (IBM Co., Armonk, NY, USA).

## Results

### In vitro study

#### Platform candidates and temperature-dependent viscosity

**Fig 1:**
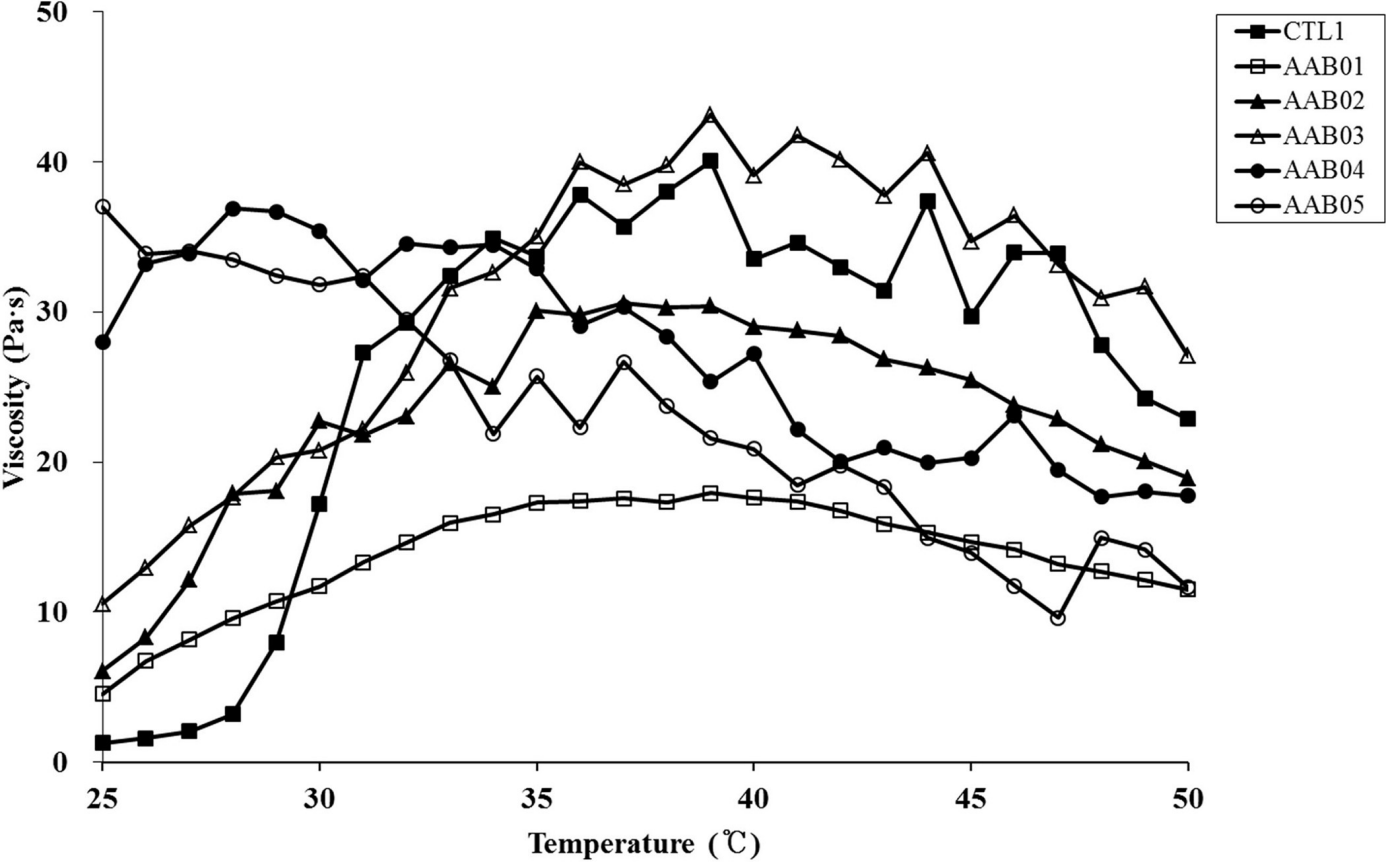
Temperature-dependent changes in viscosity for five anti-adhesion barrier (AAB) candidates and commercial anti-adhesion agent 1 (CTL1).

Temperature-dependent changes in the viscosity of the five AAB candidates (AAB01, AAB02, AAB03, AAB04, and AAB05) and CTL1 were assessed to identify the optimal composition. The results showed that AAB01 and AAB02 tended to be lower in viscosity at around 37°C than CTL1. Additionally, AAB04 and AAB05 did not have temperature sensitivity. Only AAB03 was in sol phase at room temperature (25°C), showing a gel phase in the vicinity of 37°C, and was close to the rheological properties of CTL1. Thus, we chose AAB03 as the final candidate because its change in viscosity was the most stable and similar to that of CTL1 (Table 1, Fig 1).

#### In vitro gel stability test

**Fig 2:**
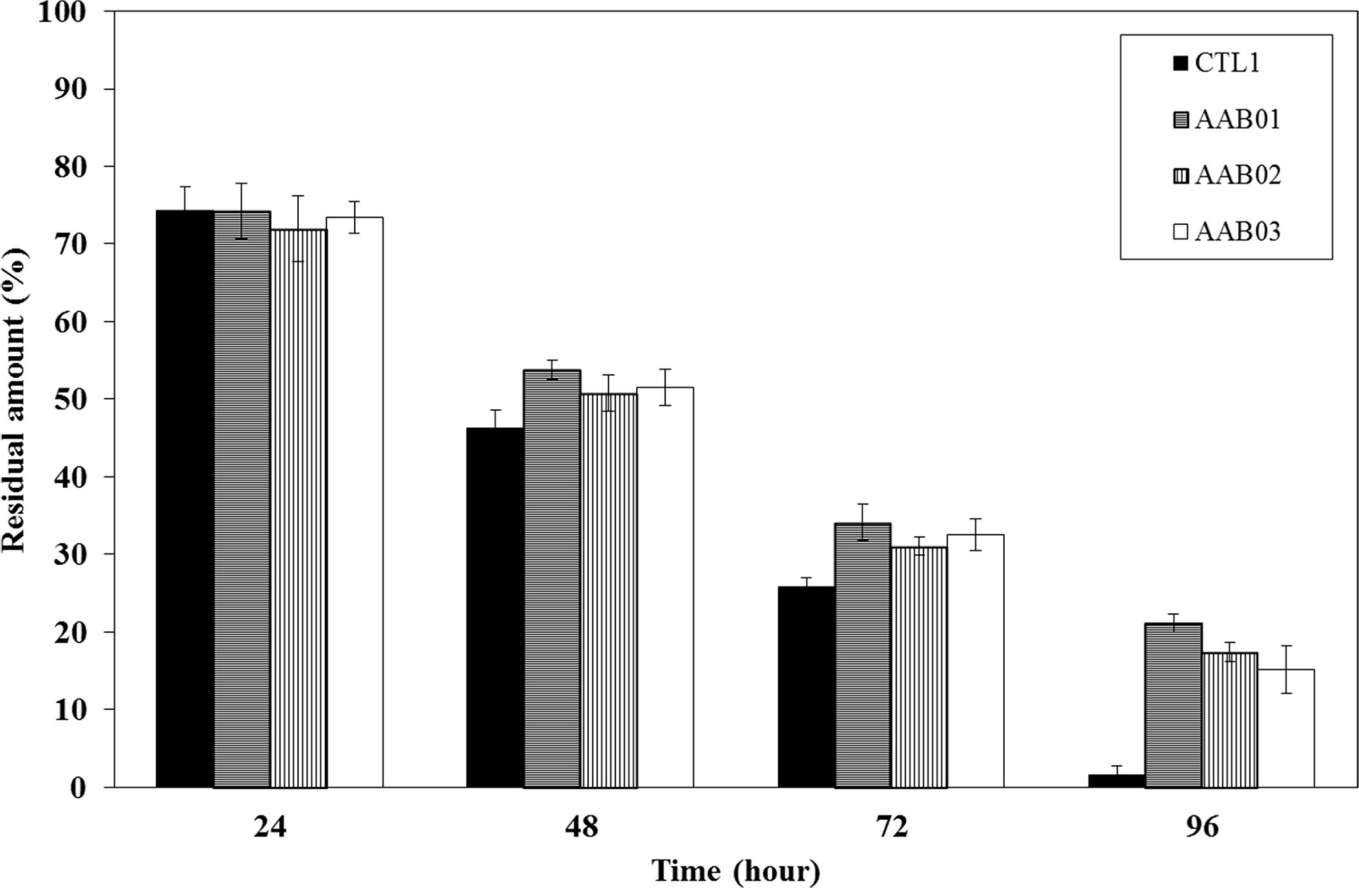
In vitro gel stability test in three selected AAB candidates (AAB01, AAB02, and AAB03) compared to that for commercial anti-adhesion agent 1 (CTL1). The AAB candidates were approximately 70% dissolved by day 4, indicating a higher level of stability than that of CTL1, which was 98% dissolved by day 4.

We performed a gel stability test on CTL1 and samples AAB01, AAB02, and AAB03, which were selected after the thermosensitivity and composition assessment. The results indicated that the water-soluble polymers in the AAB samples were converted to sol by the RPMI media, thereby reducing the amount of residual gel in all groups. Thus, these AAB candidates showed a higher level of gel stability than that for CTL1 (Fig 2).

#### Cytotoxicity test (ISO 10993–5: Extraction method and agar diffusion test)

**Fig 3:**
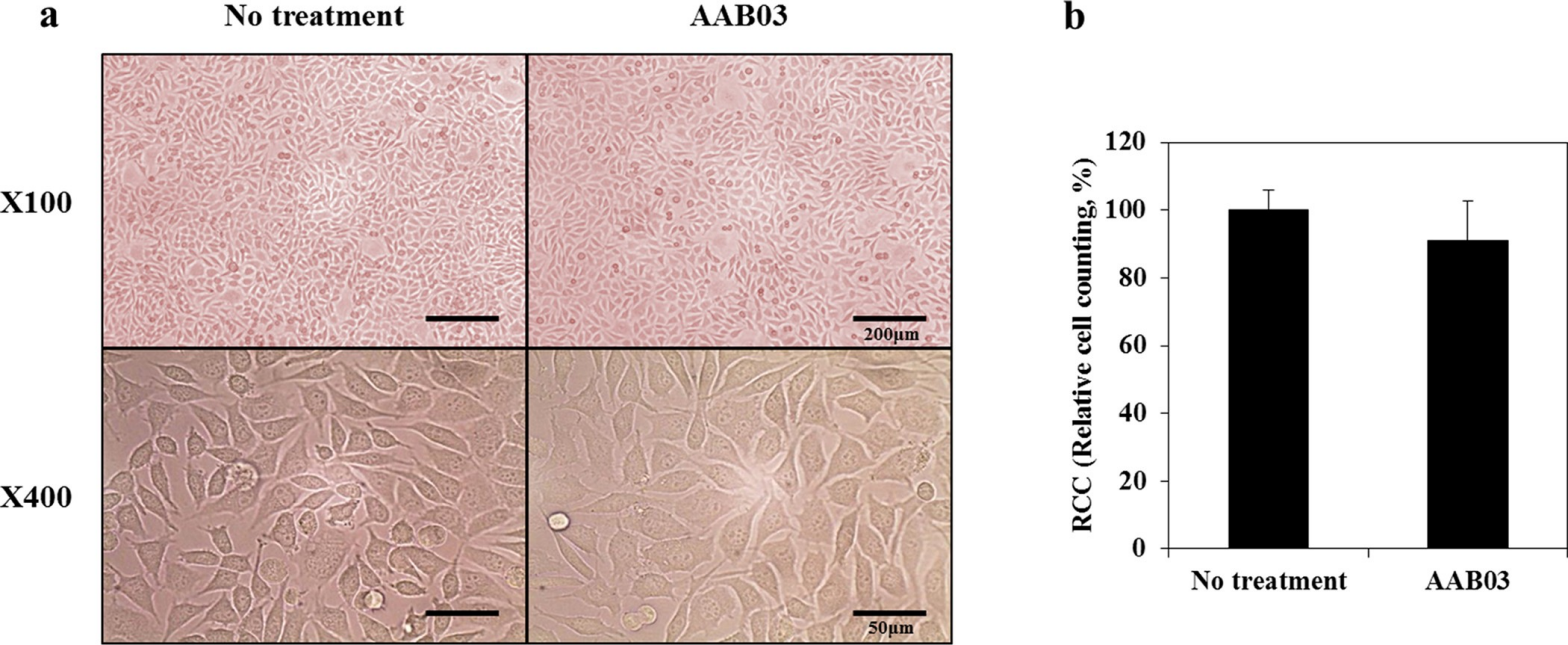
ISO 10993–5 determination of cytotoxicity. (a) Comparison of the viability and morphology of L929 fibroblast cell seeded in control and AAB03 samples after 48 hours using the ISO 10993–5 standard protocol. When employing AAB03, less than 20% of the seeded cells became round, lost intracytoplasmic granules, or showed morphologic changes. Few cells were dissolved and there was a mild growth disturbance. (b) The relative cell count (RCC) was 90.97%, which was higher than the grade 2 criteria (50%) for medical devices in the ISO 10993–5 description.

Per ISO 10993–5 specifications [25], we treated the L-929 cell line with AAB03 eluate and observed the cells after 48 hours. The relative cell count was 90.97%, which is above grade 2 criteria (50%) for medical devices, confirming that the tested device is not cytotoxic (Fig 3). The agar diffusion test also met grade 2 criteria, again confirming that the tested medical device is not cytotoxic.

### In vivo study

#### Assessment of anti-adhesive effects

**Fig 4:**
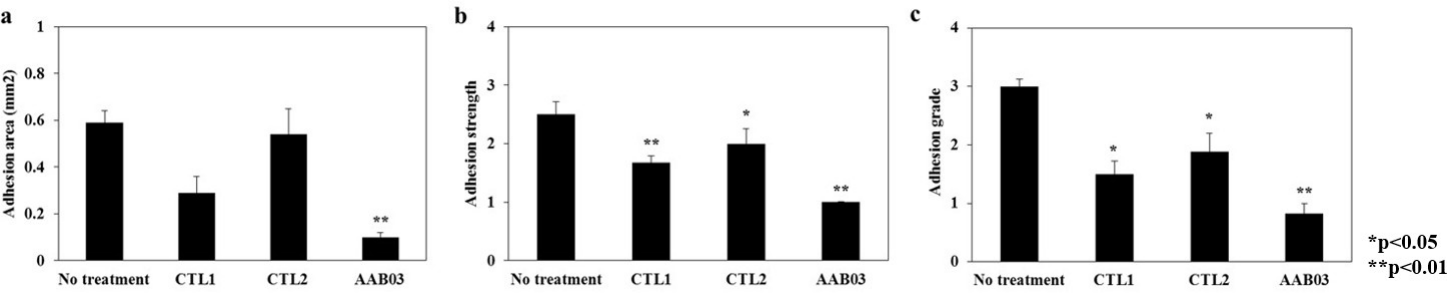
Comparisons of the adhesion area (a), strength (b), and grade (c) among no treatment (n = 10), commercial anti-adhesion agents 1 (CTL1, n = 10) and 2 (CTL2, n = 10), and AAB03 (n = 10). AAB03 is statistically superior to the control groups in terms of adhesion area, strength, and grade. *p < 0.05, **p < 0.01.

The adhesion area was 0.59 ± 0.05 mm^2^ for the no treatment group, 0.54 ± 0.11 mm^2^ for the CTL2 group, 0.29 ± 0.07 mm^2^ for the CTL1 group, and 0.10 ± 0.02 mm^2^ for the AAB03 group, with statistically significant differences among the groups (Fig 4A). The mean adhesion strength was 2.50 ± 0.22 for the no treatment group, 2.00 ± 0.25 for the CTL2 group, 1.67 ± 0.12 for the CTL1 group, and 1.00 ± 0.01 for the AAB03 group, with statistically significant differences among the groups (Fig 4B). Using Hooker’s scoring system, the mean adhesion grade was 2.83 ± 0.17 for the no treatment group, 2.17 ± 0.31 for the CTL2 group, 1.83 ± 0.17 for the CTL1 group, and 1.25 ± 0.17 for the AAB03 group, with statistically significant differences among the groups. Using the Blauer’s scoring system, the mean adhesion grade was 3.00 ± 0.13 for the no treatment group, 1.83 ± 0.31 for the CTL2 group, 1.50 ± 0.22 for the CTL1 group, and 0.83 ± 0.17 for the AAB03 group, with statistically significant differences among the groups (Fig 4C). Thus, AAB03 was statistically superior to the CTL2 and CTL1 groups in terms of adhesion area, strength, and grade.

#### Immunohistochemical analysis

**Fig 5:**
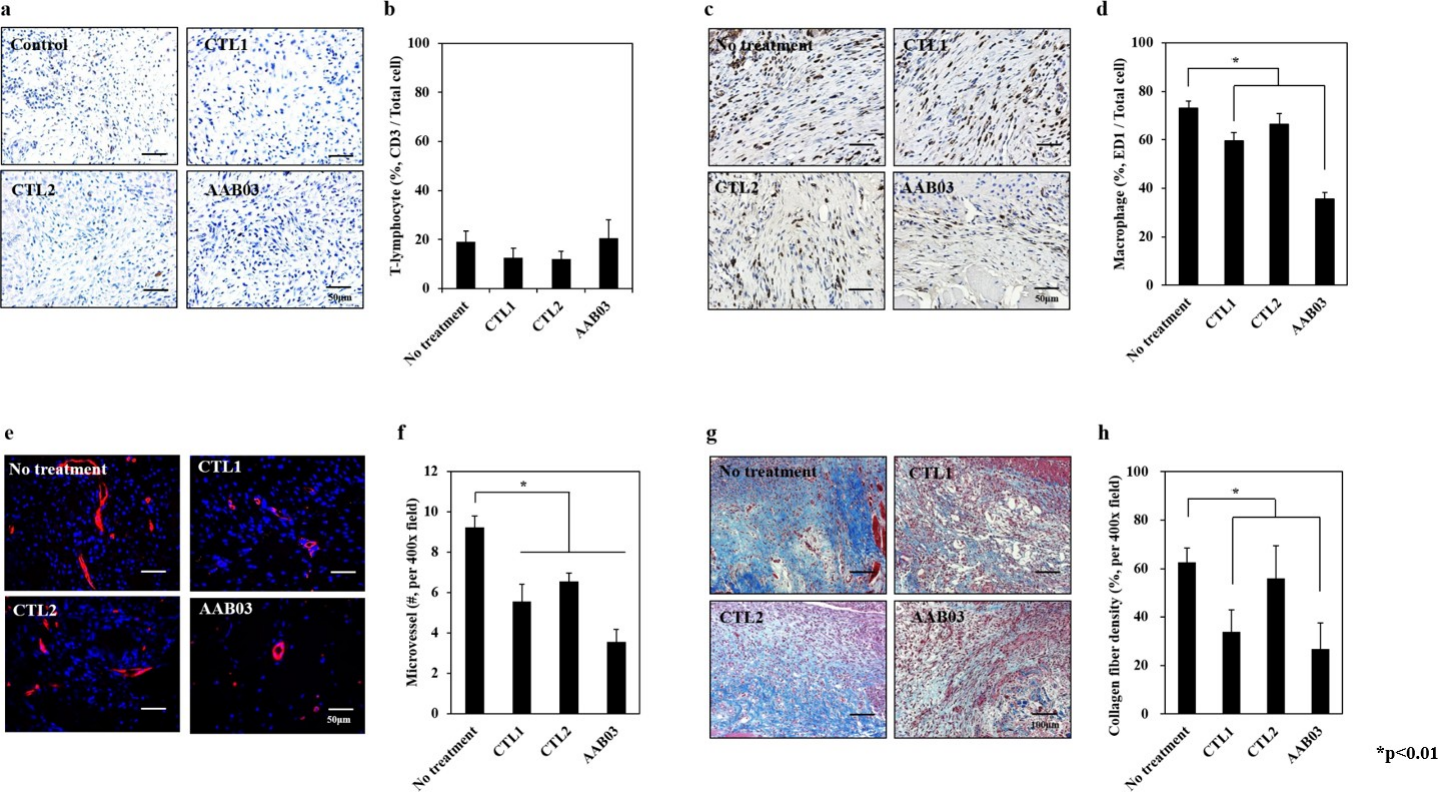
Immunohistochemical staining for the identification of the factors related to adhesion. (a) Immunohistochemical staining for CD3 (magnification x 200); (b) comparison of lymphocyte counts; (c) immunohistochemical staining for ED-1 (magnification x 200); (d) comparison of macrophage counts; (e) immunohistochemical staining for CD34 (magnification x 200); (f) comparison of microvessel density; (g) immunohistochemical staining for Masson’s trichrome (magnification x 200); (h) comparison of the collagen fiber density among no treatment (n = 10), commercial anti-adhesion agents 1 (CTL1, n = 10) and 2 (CTL2, n = 10), and AAB03(n = 10). AAB03 exhibited significantly lower macrophage counts, microvessel formation, and collagen fiber density than CTL1 and CTL2. *p < 0.01.

The lymphocyte count was 19.08 ± 4.30 for the no treatment group, 12.45 ± 3.18 for the CTL2 group, 12.00 ± 4.07 for the CTL1 group, and 20.65 ± 7.44 for the AAB03 group (Fig 5A and 5B), without statistically significant differences among the groups.

The macrophage count by ED1 was 73.11 ± 2.98 for the no treatment group, 66.44 ± 4.35 for the CTL2 group, 59.67 ± 3.29 for the CTL1 group, and 35.56 ± 2.71 for the AAB03 group, indicating that AAB03 yielded the lowest macrophage count (Fig 5C and 5D). The macrophage count by F4/80 staining did not reveal statistically significant differences among the groups (S4 Fig).

The microvessel formation score was 9.22 ± 0.58 for the no treatment group, 6.56 ± 0.41 for the CTL2 group, 5.56 ±0.85 for the CTL1 group, and 3.56 ±0.60 for the AAB03 group, indicating that AAB03 yielded the lowest level of microvessel formation (Fig 5E and 5F).

Collagen fiber density was the lowest in the no treatment group, followed by the CTL2, CTL1, and AAB03 groups (Fig 5G and 5H).

#### Systemic toxicity test

Histological examination of the heart, liver, spleen, lung, and kidneys for each group revealed no adverse events, such as inflammatory cell infiltration or cell/tissue necrosis, in the experimental and control group. (S2 Fig.)

#### Assessment of absorption and decomposition

Absorption assessment performed 7 days after injection showed that CTL1 and CTL2 were both completely absorbed in all 10 rats, but residual amounts of AAB03 were detected in 7 of 10 rats. At week 4, AAB03 was completely absorbed in 8 rats, and at week 6, AAB03 was completely absorbed in all rats (S5 Fig and S6 Fig).

## Discussion

Although the mechanism underlying adhesions is yet to be elucidated, the process begins with increased vascular permeability in the submesothelial layer, fluid leakage, and inflammatory response to tissue injury. Inflammatory cells secrete pro-inflammatory cytokines, and thrombin formed as a result of the hemostatic response converts fibrinogen into fibrin. Fibrin regenerates the injured mesothelial layer as part of normal adaptation, but during this process, fibrin attaches to surrounding organs because of its sticky properties [26]. Fibrin is typically only activated within 4–5 days of injury, which is the normal healing period. Subsequently, fibrolysis is induced by plasmin converted by tissue and urokinase plasminogen activators, leading to fibrin breakdown [27]. However, if fibrolysis is hindered for some reason until 5–7 days after injury, the presence of a fibrin matrix serves as a cause of adhesion to the surrounding organs [28]. As the fibrin matrix matures, it forms a cellular structure exhibiting vessels and nerves beyond a simple connective tissue [29].

Postoperative adhesions are the most common cause of morbidity after surgical procedures. Although adhesion formation is a physiologically ineluctable and important part of wound healing, undesirable postoperative adhesions sometimes cause serious complications, such as pain, functional intestinal obstruction, and complicated repeat surgeries. Prevention of postoperative adhesions is an important factor in reducing postoperative complications, and it requires careful surgical procedures and the use of anti-adhesive agents.

The ideal conditions of anti-adhesive agents include remaining at the wound region for at least 7 days to ensure adequate adhesion preventive effects, as the minimum time required for wound healing is 7 days (although this may vary according to wound severity). Furthermore, anti-adhesive agents should prevent fibrous tissue formation with tissues adjacent to the wound, and allow natural decomposition, absorption, or removal thereafter. Excellent adhesiveness is needed so that the anti-adhesive agents can adhere continuously to the wound region during surgery, ensuring adhesion-preventive functions [30,31]. In addition, anti-adhesive agents should be in injectable form so that they are suitable for minimal invasive surgery and laparoscopic/robotic surgery [32].

Currently, human ADM (MegaDerm^®^) is used in the form of membranes in thyroid surgery and other surgeries without any particular side effects. Human ADM is derived from donated human skin supplied by US tissue banks under the guidelines of the American Association of Tissue Banks and the US Food and Drug Administration. Donor medical history and results of serological testing were reviewed by the medical director, and fresh human cadaver skin was procured by tissue banks in accordance with these guidelines. Epidermal and dermal cells were removed without damage to essential biochemical and structural components, including collagen, elastin, and proteoglycans. The remaining acellular, dermal layer was preserved using a proprietary freeze-drying method, which retains the native extracellular architecture and vascular channels. E-beam irradiation was used to cross-link collagen and eliminate viruses, bacteria, and spores to achieve a sterility assurance level of 10^−6^ [20].

However, as mentioned above, there are some problems associated with membrane-type anti-adhesive agents. This study was designed to develop an ideal anti-adhesion agent by addressing the shortcomings of existing anti-adhesion agents. We aimed to develop a human tissue-based thermosensitive anti-adhesion agent primarily consisting of micronized ADM, hyaluronic acid, which is a vivo-derived polymer, and temperature-sensitive and biocompatible-synthesized polymers. In addition, we investigated whether the developed agent could maintain gel form within the body after injection as a liquid, where the micronized ADM forms a thin physical barrier to effectively prevent adhesion in the injury area. We investigated whether we could ensure the safety of the product by inhibiting inflammatory, foreign body, or immune responses as far as possible. We compared the properties of the developed product with those of CTL1 and CTL2, which are commercial thermosensitive anti-adhesion agents based on poloxamer/alginate widely used in South Korea.

According to the literature [20,21,33,34], ADM contains collagen and elastin, which contribute tensile strength and elasticity; proteoglycans, which induce angiogenesis; laminin, which maintains binding with connective tissues; and basement membrane, which consists of collagen type IV. This material acts as a biologic scaffold for re-epithelialization, neovascularization, and fibroblast infiltration but does not induce an immune response. ADM creates a collagen-rich scaffold and provides long-term stem cell differentiation, tissue regeneration, and revascularization within the host. Cellular infiltration, primarily by macrophages, is initiated after ADM implantation. In addition, an increase in neovascularization and fibrosis has been observed within ADM.

In our previous study [20], the expression of proteins involved in wound healing and remodeling, such as collagen type 1, transforming growth factor-b, and matrix metalloproteinases, was higher than that in a control group. In addition, a histologic analysis revealed high cellular infiltration at 1 month after ADM implantation and the deposition of collagen and elastin fibers was increased at 6 months compared to that at 1 month after implantation. These results suggest a rapid cellular infiltration of the implanted ADM but slower and controlled extracellular matrix (ECM) remodeling, leading to long-term structural integrity and increased durability. Additionally, microvessel density increased at 3 and 6 months compared to that at 1 month after ADM implantation, indicating long-term remodeling of the ECM in implanted ADM.

Based on the clinical data for wound healing and remodeling in ADM, we conducted a randomized, controlled trial comparing ADM implantation versus no ADM implantation during open total thyroidectomy [21]. Patients with ADM implantation showed a better outcome with regard to the objective severity of the postoperative scar. Participants in the group with ADM had lower Vancouver scar scale scores and a lesser degree of both pigmentation and postoperative erythema than those in the control group. Moreover, no patients in the ADM implantation group showed any postoperative hypertrophy within 2 months after surgery [21].

In the present study, we tested the temperature-dependent viscosity of several AAB samples and chose AAB03 [poloxamer 127 18.0wt%, hyaluronic acid 0.2% (w/v), micronized ADM 1.75% (w/v)] as the final candidate because it exhibited similar properties to those of CTL1 and was the most stable candidate. The sample remained in a less viscous sol state at room temperature (25°C) but solidified to gel at approximately body temperature (35°C); therefore, it could provide an effective barrier in areas with potential adhesion.

Cytotoxicity testing of the AAB03 sample confirmed that AAB03 is not cytotoxic, as it achieved grade 2 according to ISO 10993–5 specifications and the agar diffusion test. Animal testing also confirmed that AAB03 is safe for use on major organs.

Animal testing of adhesion resistance confirmed that AAB03 is statistically superior to no treatment and treatment with CTL1 or CTL2 in terms of adhesion area, strength, and grade. Adhesion was assessed using a double-blinded method. However, to adjust for experimental errors caused by the subjective decisions of the examiner and to maintain the objectivity of the experiment, we used specific staining methods to observe factors related to adhesion formation, namely, lymphocytes, macrophages, microvessels, and collagen fiber. The results confirmed that AAB03 exhibited significantly lower lymphocyte and macrophage counts and lower microvessel density than CTL1 and CTL2. An MT stain showed that AAB03 also exhibited lower collagen fiber density. These results were consistent with the adhesion resistance results. We believe that AAB03 could be an effective anti-adhesion agent because it prevents infiltration of lymphocytes, macrophages, microvessels, and collagen fiber.

One of the properties of an ideal anti-adhesion agent is the ability for the substance to remain an effective barrier for at least 7 days, after which it must be completely absorbed within the body. An absorption assessment showed that CTL1 and CTL2 were both completely absorbed in all rats by day 7, although residual amounts of AAB03 were detected in 7 of 10 rats. AAB03 was completely absorbed in all rats by week 6. Because of the mechanism of scar fibrosis, the process continues until about 1 month; the conventional anti-adhesion agent dissolves and disappears within 7 days, whereas AAB03 lasts longer than 1 month and is thought to promote scar remodeling as well as scar fibrosis.

## Conclusions

The micronized ADM-based thermosensitive anti-adhesion agent, AAB03, developed in the present study appears to be an ideal anti-adhesion agent incorporating the strengths of existing gel- and membrane-type anti-adhesion agents. If the similar outcomes manifest in clinical settings, AAB03 could be used as a superior postoperative anti-adhesion agent over current anti-adhesive products, improving the patient quality of life.

## Disclosures

All authors confirm that there are no known conflicts of interest associated with this paper and there has been no significant financial support for this work that could have influenced its outcome.

## Supporting information

S1 Fig Rat models of abdominal sidewall defects, cecum abrasions, absorption, and surgical procedures. CTL1: commercial anti-adhesion agent 1, CTL2: commercial anti-adhesion agent 2, AAB03: anti-adhesion barrier 03.
(TIF)

S2 Fig Histological findings of the brain, heart, kidney, liver, and spleen at 7 days postsurgery. Abnormal features, such as inflammatory responses and cell or tissue necrosis, were not noted for control and experimental groups. Moreover, histological observation showed no significant differences among the groups in the examined organ tissues (H& E stain, x 200).
Control: No treatment, AAB03: anti-adhesion barrier 03.
(TIF)

S3 Fig Anti-adhesion effects at 7 days postsurgery. Macroscopic observation at postoperative day 7 shows that anti-adhesion barrier 03 (AAB03) has superior anti-adhesion effects to those of no treatment and treatment with commercial anti-adhesion agents 1 (CTL1) and 2 (CTL2).
(TIF)

S4 Fig Immunohistochemical staining with ED1 and F4/80 for macrophages. Macrophages were counted using an optical microscope after immunostaining with ED1 and F4/80 antibodies (dilution = 1:100 and 1:50). There were no statistically significant differences among No treatment (n = 10), CTL1 (n = 10), CTL2 (n = 10), and AAB03 (n = 10) groups.
CTL1: commercial anti-adhesion agent 1, CTL2: commercial anti-adhesion agent 2, AAB03: anti-adhesion barrier 03.
(TIF)

S5 Fig Comparison in the residual material at postoperative day 7 among commercial anti-adhesion agents 1 (CTL1) and 2 (CTL2) and anti-adhesion barrier 03 (AAB03) groups. (a) Gross findings: no residual anti-adhesion agent material in the CTL1 and CTL2 groups. (b) Absorption rate: residual amounts of AAB03 were detected in 7/10 rats.
(TIF)

S6 Fig Assessment of absorption and decompression. Anti-adhesion barrier 03 (AAB03) was completely absorbed in eight rats at 4 weeks and in all rats at 6 weeks. (a) Gross findings. (b) Residual volume.
(TIF)
