# Supplementary figures and images for: Development of novel biocompatible thermosensitive anti-adhesive agents using human-derived acellular dermal matrix
Source: PLoS One. 2019 Feb 22;14(2):e0212583. doi: 10.1371/journal.pone.0212583 (PMC6386309; doi:10.1371/journal.pone.0212583)

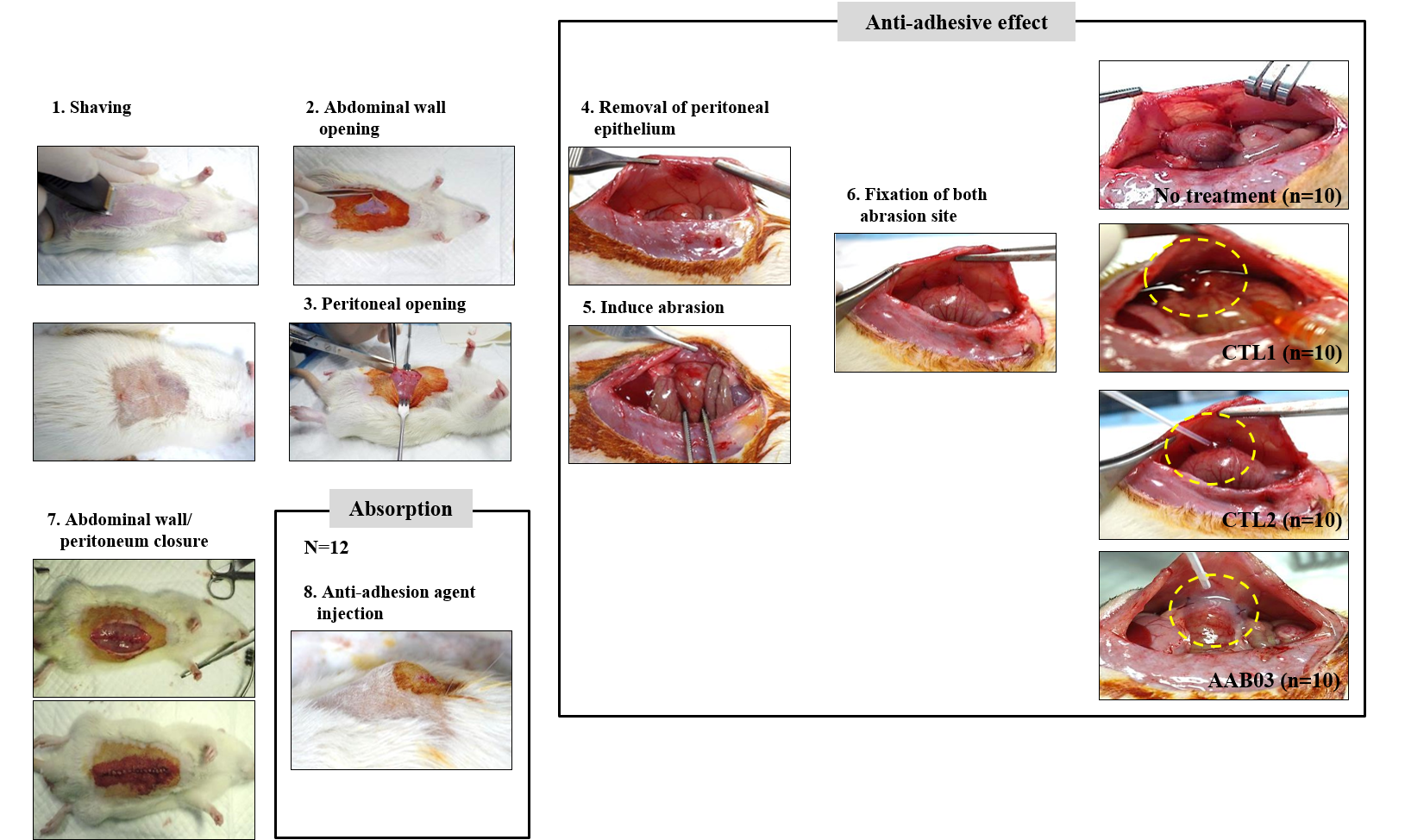

Supplement: S1 Fig — CTL1: commercial anti-adhesion agent 1, CTL2: commercial anti-adhesion agent 2, AAB03: anti-adhesion barrier 03. (TIF) [file pone.0212583.s001.tif]

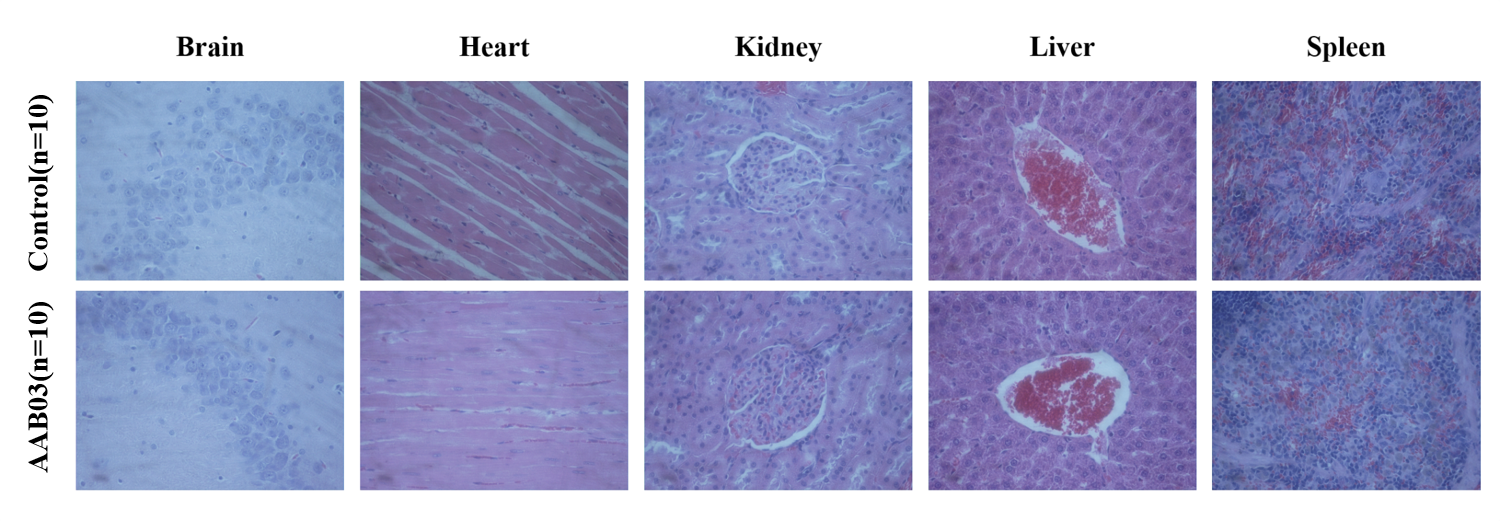

Supplement: S2 Fig — Abnormal features, such as inflammatory responses and cell or tissue necrosis, were not noted for control and experimental groups. Moreover, histological observation showed no significant differences among the groups in the examined organ tissues (H& E stain, x 200). Control: No treatment, AAB03: anti-adhesion barrier 03. (TIF) [file pone.0212583.s002.tif]

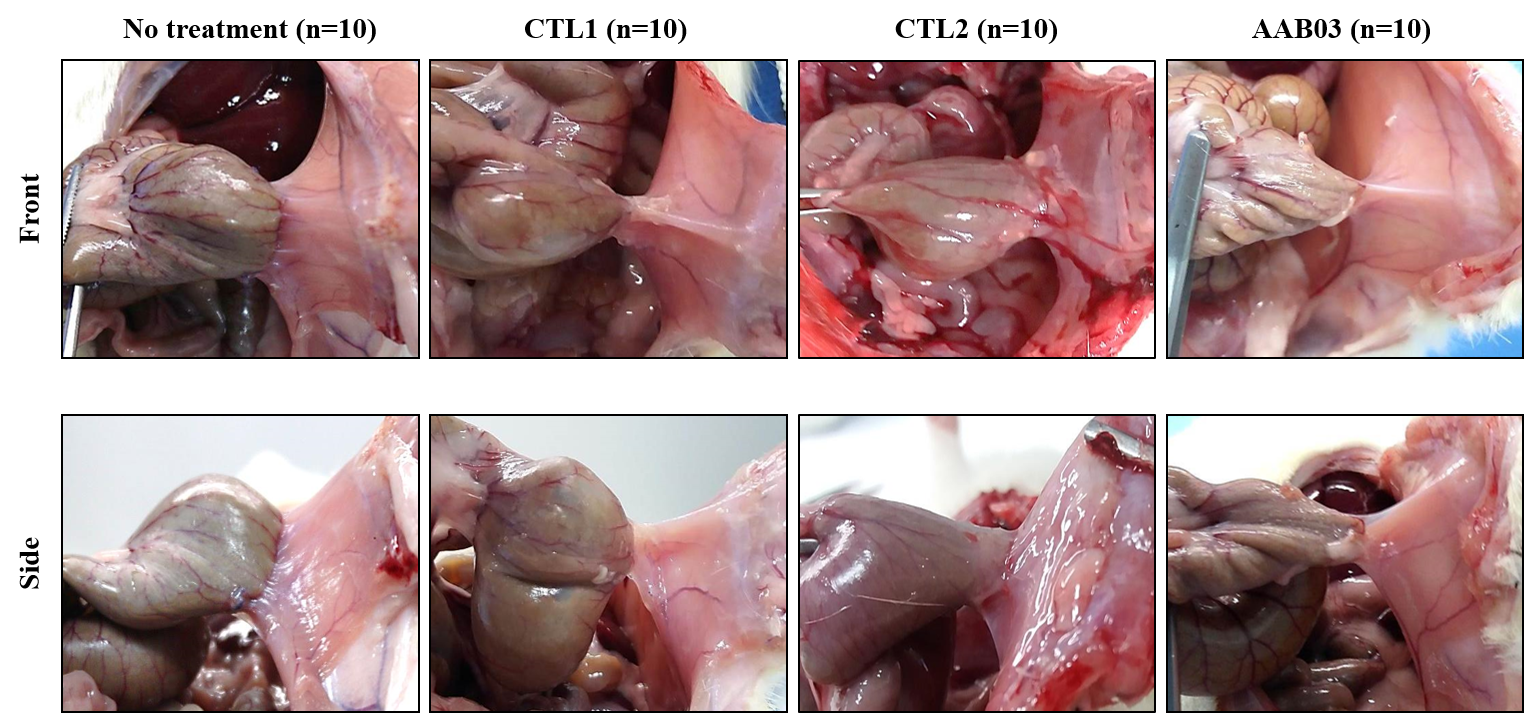

Supplement: S3 Fig — Macroscopic observation at postoperative day 7 shows that anti-adhesion barrier 03 (AAB03) has superior anti-adhesion effects to those of no treatment and treatment with commercial anti-adhesion agents 1 (CTL1) and 2 (CTL2). (TIF) [file pone.0212583.s003.tif]

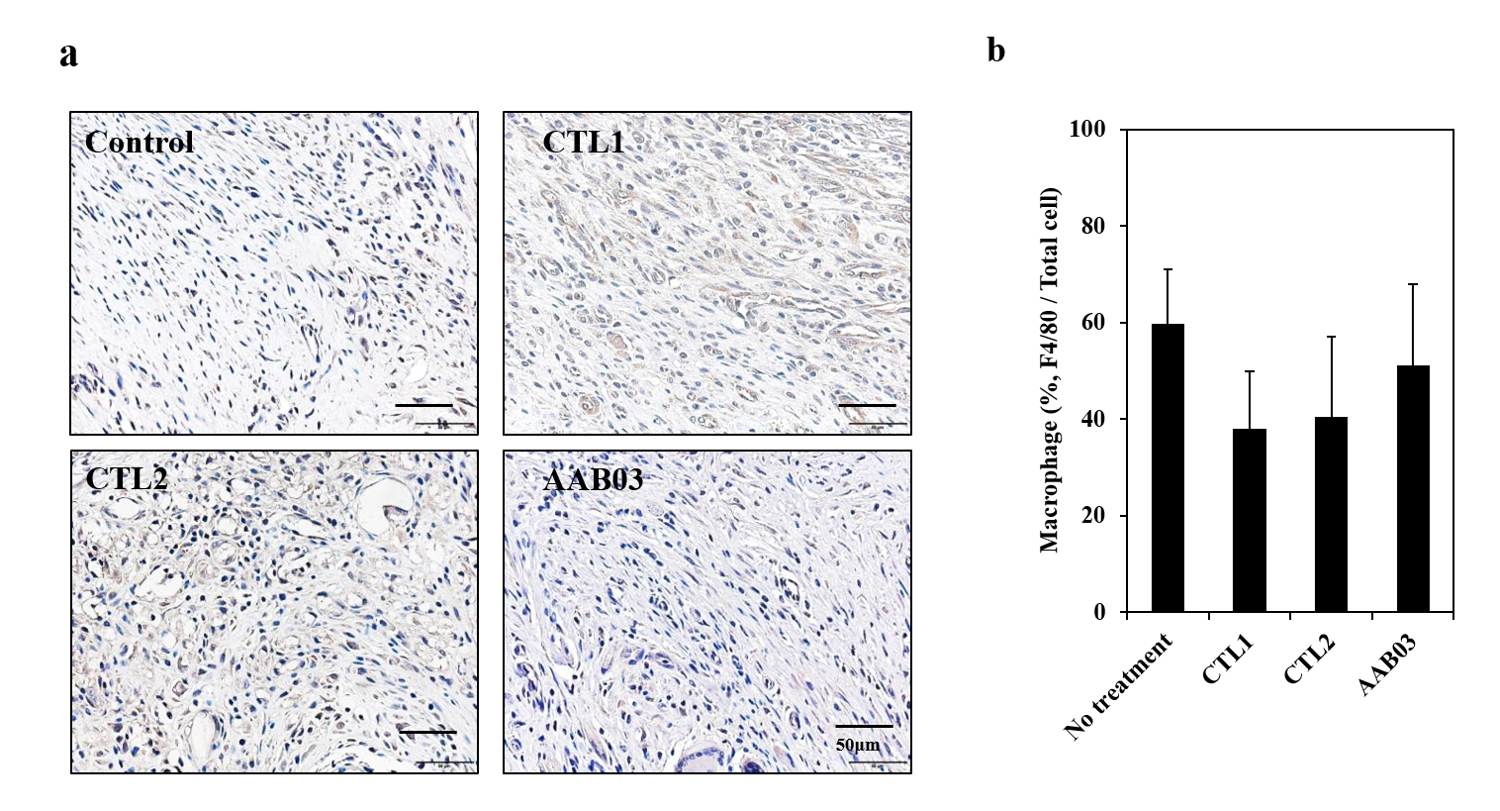

Supplement: S4 Fig — Macrophages were counted using an optical microscope after immunostaining with ED1 and F4/80 antibodies (dilution = 1:100 and 1:50). There were no statistically significant differences among No treatment (n = 10), CTL1 (n = 10), CTL2 (n = 10), and AAB03 (n = 10) groups. CTL1: commercial anti-adhesion agent 1, CTL2: commercial anti-adhesion agent 2, AAB03: anti-adhesion barrier 03. (TIF) [file pone.0212583.s004.tif]

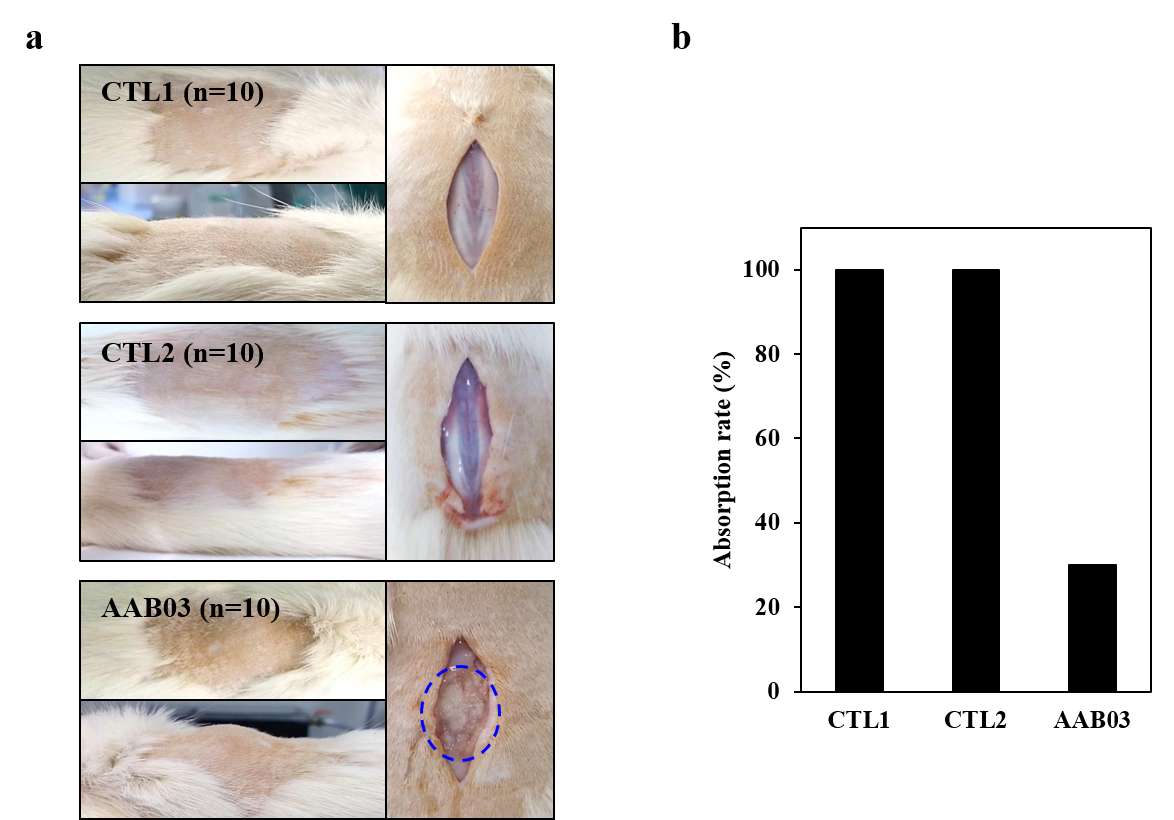

Supplement: S5 Fig — (a) Gross findings: no residual anti-adhesion agent material in the CTL1 and CTL2 groups. (b) Absorption rate: residual amounts of AAB03 were detected in 7/10 rats. (TIF) [file pone.0212583.s005.tif]

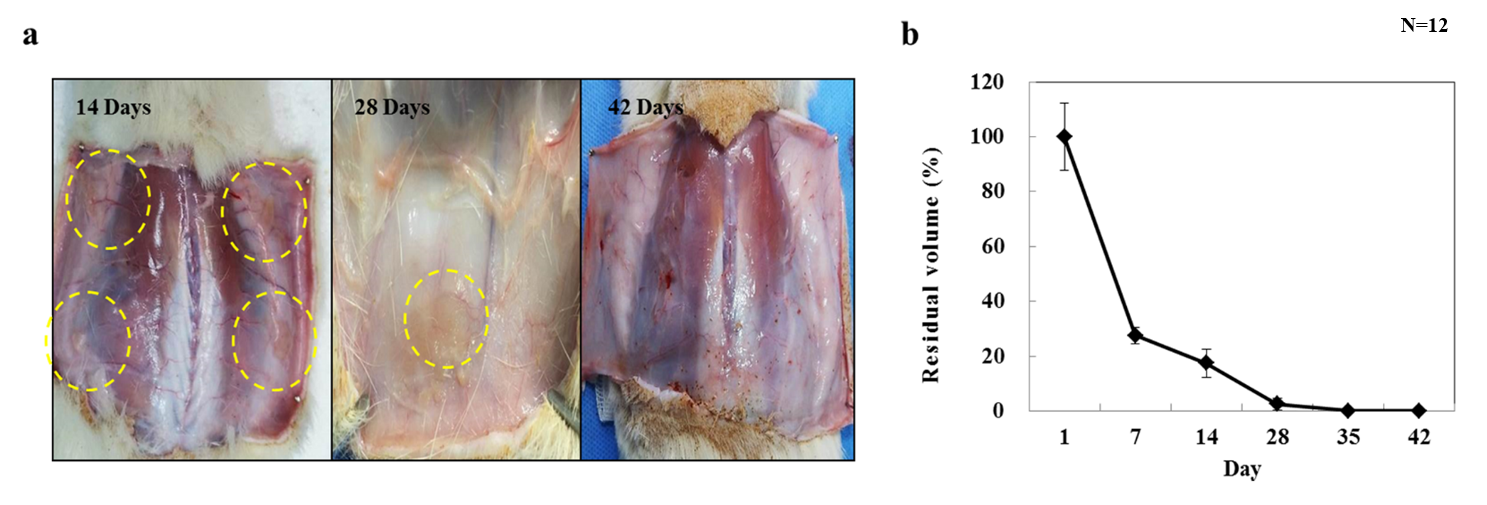

Supplement: S6 Fig — Anti-adhesion barrier 03 (AAB03) was completely absorbed in eight rats at 4 weeks and in all rats at 6 weeks. (a) Gross findings. (b) Residual volume. (TIF) [file pone.0212583.s006.tif]
